# A novel classification for medial malleolar fracture based on the 3-D reconstruction CT

**DOI:** 10.1186/s13018-021-02688-9

**Published:** 2021-08-28

**Authors:** Fangke Hu, Guoyun Bu, Jun Liang, Haijing Huang, Jinquan He

**Affiliations:** 1 Orthopedic Department, Tianjin Hospital, Tianjin, 300211 China; 2 Orthopedic Trauma Department of Foot and Ankle Surgery I, Tianjin Hospital, Tianjin, 300211 China

**Keywords:** Medial malleolus, Fracture, 3-D reconstruction CT, Classification

## Abstract

**Background:**

Fracture of the medial malleolus is one of the most frequent injuries treated surgically; however, the classification of the fracture has not attracted much attention and a good classification system is still lacking.

**Methods:**

Consecutive cases of medial malleolus fractures were prospectively enrolled. Based on the 3-D reconstruction CT morphology and centered on the posterior colliculus of the medial malleolus, we classified the fractures into 4 types: type 1 with no involvement of the posterior colliculus, type 2 with partial involvement of posterior colliculus, type 3 with the entire involvement of posterior colliculus, and type 4 with the fracture line 4 vertically extended from the intercollicular groove to the comminuted fracture of the posterior malleolus. Statistical analyses were performed to evaluate the clinical significance of the classification.

**Results:**

There were 273 cases prospectively enrolled. The distribution of the cases was type 1 of 12.1%, type 2 of 41.0%, type 3 of 30.0%, and type 4 of 16.8%. Statistics showed that the new classification had significant associations but did not totally depend on the classical ankle fracture classifications. Results showed that the new classification had implications in the severity of ankle fractures. From type 1 to type 4, the ankle joint was more and more unstable. Furthermore, comminuted medial malleolar fractures could be subdivided, and the new classification could provide useful information for surgical decision-making.

**Conclusions:**

The novel classification was a useful system to describe the 3-D geometry of the fractured medial malleolus.

**Supplementary Information:**

The online version contains supplementary material available at 10.1186/s13018-021-02688-9.

## Introduction

Fracture of the medial malleolus is one of the most frequent injuries treated by the orthopedic surgeon [1]. Either in isolation or in conjunction with fractures of the lateral and posterior malleolus, displaced medial malleolar fracture has been recommended for operative management [2, 3]; however, the classification of the fracture has not been paid much attention by the orthopedic surgeon.

Fractures of the ankle joint are commonly classified according to the Danis-Weber [4–6] and Lauge-Hansen systems [7]. However, all the two classifications were centered on the injury of the lateral malleolus, and the fracture morphology of medial malleolus was little mentioned [8]. There have been attempts at introducing classification systems for the medial malleolar fracture. The Pankovich system [9, 10] and the modified Pankovich system [11] are based on the location of the medial malleolar fracture and the competency of the deltoid ligament and divided the fractures into 6 groups. The Ebraheim system [12, 13] is based on the level and location of the medial malleolar fracture and classified into 4 groups: transverse, oblique, comminuted, and vertical fractures. The Herscovici system [14] is based on the level of medial malleolar fracture and classified into 4 groups: avulsion fractures, between the tip and plafond, at the level of the plafond, and vertical fractures. Unfortunately, all the above classifications were mostly used for the descriptions of fracture morphology based on X-ray film while the clinical significance was unclear.

The medial malleolus is composed of the anterior colliculus, the intercollicular groove, and the posterior colliculus. In the latest years, the posterior colliculus has been considered to be the primary stabilizer of the medial ankle [1, 15]. Considering the great clinical significance of fracture line direction and the comminuted morphology for the lateral malleolus, we proposed that it also had clinical significance for the medial malleolus. According to the theory, we raised a posterior colliculus-centric classification system based on the 3-D reconstruction CT of medial malleolar fracture. The present study was performed to describe the novel classification system and to evaluate the clinical significance of the classification.

## Materials and methods

We prospectively enrolled all consecutive patients with medial malleolar fractures according to inclusion and exclusion criteria from January 1, 2018, to October 31, 2019, at our department of foot and ankle surgery. The inclusion criteria were patients with medial malleolar fractures enrolled at our department. In order to predict the association of medial malleolar fracture with lateral and posterior malleolar fractures as well as to simplify the statistical analysis, we excluded cases of isolated medial malleolar fractures and cases of avulsion malleolar fractures of the tip. Other exclusion criteria were open fractures, Pilon fractures, multiple fractures in the same foot, old fractures more than 3 weeks, pathologic fractures, fractures treated by conservative therapy, patients younger than 18 years, and patients who refused to join in the study. The 3-D reconstruction CT was routinely performed for all the patients after closed reduction and radiography was prospectively collected from the radiology department. With the approval of our institutional review board, all the information was collected in accordance with the World Medical Association Declaration of Helsinki and written consents were obtained from all the patients.

**Fig. 1:**
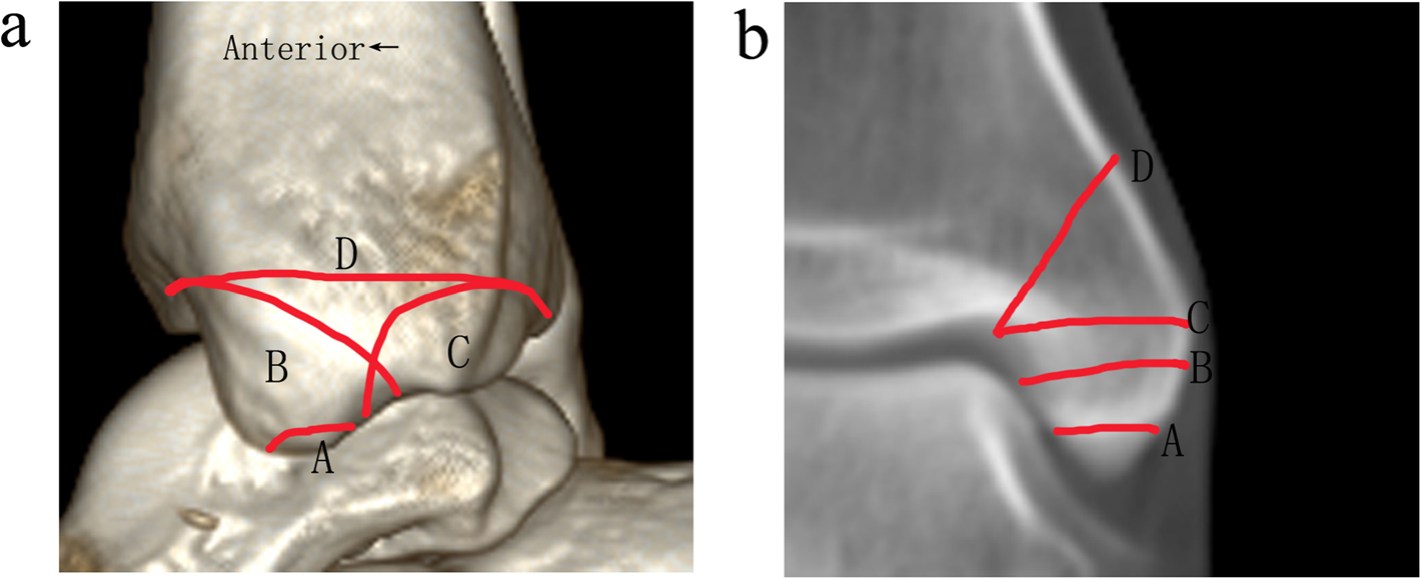
The modified Pankovich classification and the Herscovici classification to classify the medial malleolar fractures. **a** The modified Pankovich classification. **b** The Herscovici classification

All the classifications were separately judged by two senior orthopedic surgeons. If a disagreement arose, the case would be discussed with a third senior orthopedic surgeon until an agreement was reached. Ankle fractures were classified according to the Danis-Weber system (AO classification) [4, 16], the Lauge-Hansen system [7, 16], the modified Pankovich classification [11], and the Herscovici classification [14] based on the standard X-rays. As shown in Fig. 1a, the modified Pankovich classification includes 4 types of type A with no or avulsion fracture, type B with anterior colliculus fracture, type C with posterior colliculus fracture, and type D with supracollicular fracture [11]. As shown in Fig. 1b, the Herscovici system includes 4 fracture lines of type A with avulsion fracture of the tip, type B occurs between the tip and the level of the plafond, type C at the level of the plafond, and type D extends vertically above this level [14].

**Fig. 2:**
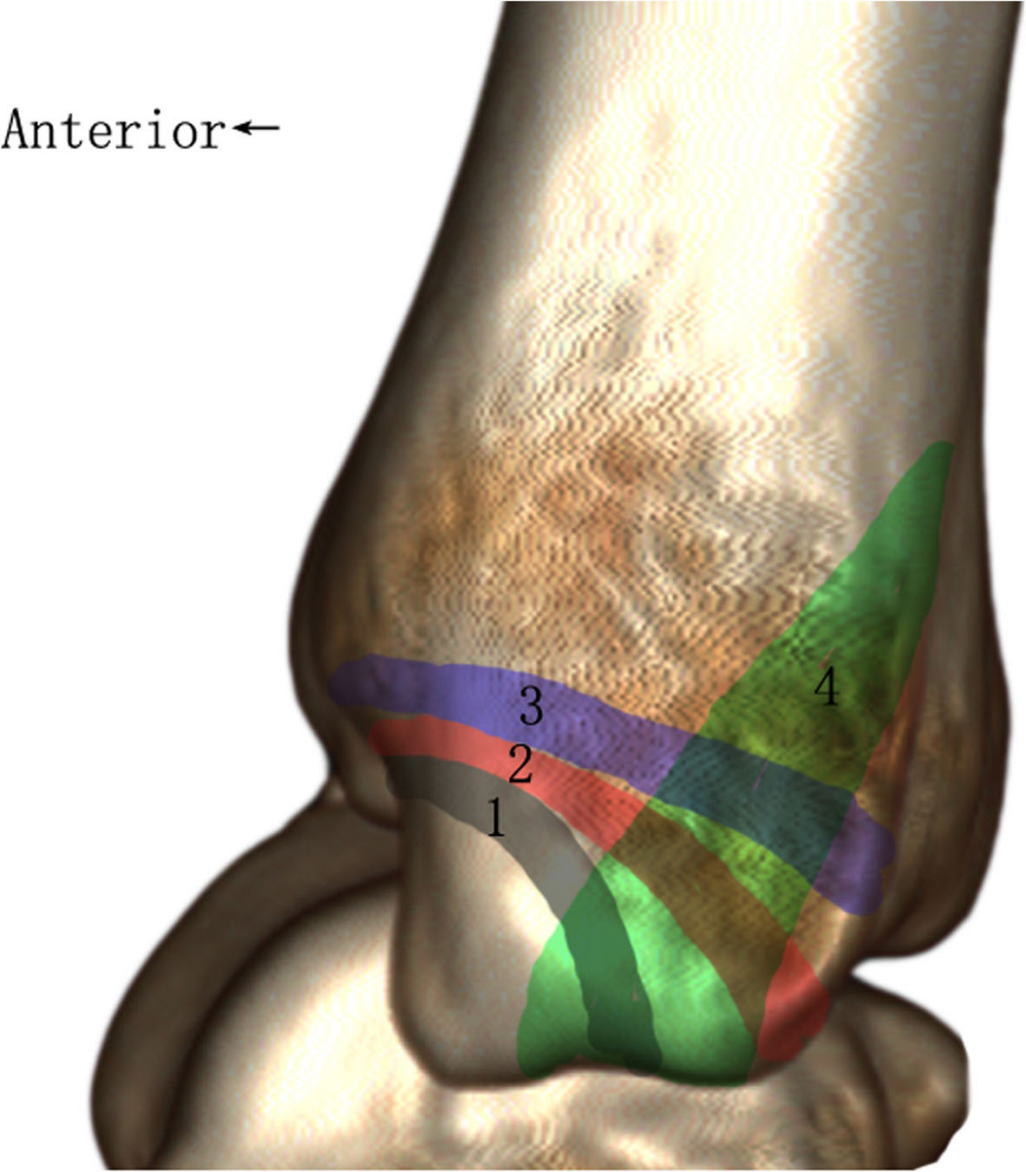
The new fracture line system to classify the medial malleolar fractures according to the 3-D reconstruction CT

Considering the importance of the posterior colliculus in the stability of medial ankle fracture, we raised a new fracture line classification centered on the integrity of the posterior colliculus (Fig. 2). Fracture line 1 (type 1) extended from the anterior of the medial malleolus to the intercollicular groove with no involvement of the posterior colliculus. Fracture line 2 (type 2) extended from the anterior of the medial malleolus to the posterior colliculus or the distal groove for posterior tibial tendon with the partial involvement of posterior colliculus. Fracture line 3 (type 3) obliquely horizontally extended from the upper anterior of the medial malleolus to the proximal groove for the posterior tibial tendon with the entire involvement of the posterior colliculus. Fracture line 4 (type 4) vertically extended from the intercollicular groove or the posterior colliculus to the distal tibia which was always combined with other 3 fracture lines with the comminuted fracture of the posterior colliculus. Notably, all the comminuted cases with fracture line 4 were included in type 4.

The presence or absence of distal tibiofibular syndesmotic injury was recorded, with the distance of syndesmosis between the tibia and fibula <4mm of no syndesmotic injury, 4–7mm of syndesmotic injury, and >7mm of syndesmotic separation measured on the mortise view. The lateral displacement of the talus was measured by the lateral border of the talus versus the lateral border of the distal tibial plafond on the coronal CT images at the middle level of the medial malleolus. The joint surface involvement of the distal tibial plafond and the angle between the major fracture line and distal tibial plafond was also measured on the coronal CT images at the middle level of the medial malleolus. The presence of Maisonneuve fractures, the separation of the anterior and posterior colliculus, and the comminuted fractures of medial malleolar were determined on the 3-D reconstruction CT radiographies. Intraoperative surgery details such as surgical approaches, fixation methods, distal tibiofibular syndesmosis repairs, and posterior malleolar managements were prospectively recorded.

Statistical calculations were performed with SPSS 18.0 software (SPSS Inc, Chicago, IL, USA). Kolmogorov-Smirnov tests were used to check Gaussian distribution for continuous variables. *T* tests were used for all the continuous variables according to Gaussian distribution. For the ranking variables, Pearson chi-square tests were used. Bivariate correlation analysis was used to identify correlations between these variables. The level of statistical significance was set at a two-sided *P*-value of 0.05.

## Results

There were 469 patients who met the inclusion criteria and 196 patients were excluded. There were 273 patients included in the final analysis. The mean age was 46.4 ± 15.7 years and the female percent was 51.3% (140/273). According to our new 3-D CT classification of medial malleolus, there were 12.1% of type 1 (fracture line 1 type), 41.0% of type 2 (fracture line 2 type), 30.0% of type 3 (fracture line 3 type), and 16.8% of type 4 (fracture line 4 co-existence type). Presented in Table 1, no difference could be found between the 4 groups in the baseline characteristics of gender, age, mechanism of injury, and number of Maisonneuve fractures. Significant differences could be found in the 4 groups of different groups of the Lauge-Hansen classification, the Danis-Weber classification, the Modified Pankovich classification, the Herscovici classification, anterior and posterior colliculus separation, comminuted medial malleolar fracture, lateral displacement of the talus, joint surface involvement of the distal tibial plafond, angle between the major fracture line and the distal tibial plafond, and intraoperative posterior malleolar management.

**Table 1:** Comparison of the characteristics among the groups of the new fracture line classification

Characteristics		Type 1 (***N*** = 33, 12.1%)	Type 2 (***N*** = 112, 41.0%)	Type 3 (***N*** = 82, 30.0%)	Type 4 (***N*** = 46, 16.8%)	Total (***N*** = 273)	Significance (***P***)
**Gender**	**Female**	14 (42.4%)	58 (51.8%)	44 (53.7%)	24 (52.2%)	140 (51.3%)	0.742
	**Male**	19 (57.6%)	54 (48.2%)	38 (46.3%)	22 (47.8%)	133 (48.7%)	
**Age (years)**		44.4 ± 17.9	45.1 ± 16.4	49.0 ± 14.2	46.1 ± 14.8	46.4 ± 15.7	0.325
**Mechanism of injury**	**Low energy**	28 (84.8%)	92 (82.1%)	59 (72.0%)	37 (80.4%)	216 (79.1%)	0.275
	**High energy**	5 (15.2%)	20 (17.9%)	23 (28.0%)	9 (19.6%)	57 (20.9%)	
**Lauge-Hansen classification**	**SE**	30 (90.9%)	90 (80.4%)	52 (63.4%)	34 (73.9%)	206 (75.5%)	0.002*
	**SA**	0 (0%)	0 (0%)	6 (7.3%)	0 (0%)	6 (2.2%)	
	**PE**	3 (9.1%)	22 (19.6%)	22 (26.8%)	12 (26.1%)	59 (21.6%)	
	**PA**	0 (0%)	0 (0%)	2 (2.4%)	0 (0%)	2 (0.7%)	
**Danis-Weber classification**	**A**	0 (0%)	2 (1.8%)	7 (8.5%)	1 (2.2%)	10 (3.7%)	0.012*
	**B**	29 (87.9%)	87 (77.7%)	50 (61.0%)	30 (65.2%)	196 (71.8%)	
	**C**	4 (12.1%)	23 (20.5%)	25 (30.5%)	15 (32.6%)	67 (24.5%)	
**Modified Pankovich classification**	**B**	32 (97.0%)	1 (0.9%)	0 (0%)	0 (0%)	33 (12.1%)	<0.001*
	**C**	1 (3.0%)	1 (0.9%)	0 (0%)	16 (34.8%)	18 (6.6%)	
	**D**	0 (0%)	110 (98.2%)	82 (100%)	30 (65.2%)	222 (81.3%)	
**Herscovici classification**	**B**	28 (38.9%)	39 (34.8%)	2 (1.1%)	3 (6.5%)	72 (26.4%)	<0.001*
	**C**	5 (15.2%)	73 (65.2%)	60 (73.2%)	14 (30.4%)	152 (55.7%)	
	**D**	0 (0%)	0 (0%)	20 (24.4%)	29 (63.0%)	49 (17.9%)	
**Anterior and posterior colliculus separation**	**No**	3 (9.1%)	108 (96.4%)	77 (93.9%)	16 (34.8%)	204 (74.7%)	<0.001*
	**Yes**	30 (90.9%)	4 (3.6%)	5 (6.1%)	30 (65.2%)	69 (25.3%)	
**Comminuted medial malleolus fracture**	**No**	28 (84.8%)	95 (84.8%)	55 (67.1%)	11 (23.9%)	189 (69.2%)	<0.001*
	**Yes**	5 (15.2%)	17 (15.2%)	27 (32.9%)	35 (76.1%)	84 (30.8%)	
**Distal tibiofibular syndesmosis injury**	**No**	26 (78.8%)	68 (60.7%)	45 (54.9%)	25 (54.3%)	164 (60.0%)	0.041*
	**Injury**	6 (18.2%)	26 (23.2%)	19 (23.2%)	17 (37.0%)	68 (24.9%)	
	**Separation**	1 (3.0%)	18 (16.1%)	18 (21.9%)	4 (8.7%)	41 (15.0%)	
**Maisonneuve fracture**	**No**	32 (97.0%)	107 (95.5%)	80 (97.6%)	45 (97.8%)	264 (96.7%)	0.836
	**Yes**	1 (3.0%)	5 (4.5%)	2 (2.4%)	1 (2.2%)	9 (3.3%)	
**Lateral displacement of talus (mm)**		6.9 ± 4.1	8.6 ± 5.8	9.0 ± 5.8	5.1 ± 3.4	7.9 ± 5.4	<0.001*
**Joint surface involvement of distal tibial plafond**	**No**	33 (100%)	106 (94.6%)	55 (67.1%)	17 (37.0%)	211 (77.3%)	<0.001*
	**Yes**	0 (0%)	6 (5.4%)	27 (32.9%)	29 (63.0%)	62 (22.7%)	
**Angle between the major fracture line and distal tibial plafond (degree)**		6.6 ± 12.1	16.3 ± 13.3	37.2 ± 17.0	55.0 ± 14.8	26.3 ± 21.0	<0.001*
**Intraoperative surgical approach**	**Anterior- inferior**	33 (100%)	112 (100%)	82 (100%)	35 (76.1%)	262 (96.0%)	<0.001*
	**Posterior-medial**	0 (0%)	0 (0%)	0 (0%)	11 (23.9%)	11 (4.0%)	
**Intraoperative medial malleolus fixation method**	**Lag screw**	32 (97.0%)	109 (97.3%)	75 (91.5%)	25 (54.3%)	239 (88.3%)	<0.001*
	**Buttress plate**	0 (0%)	0 (0%)	5 (6.1%)	16 (34.8%)	23 (7.7%)	
	**K-wire**	1 (3.0%)	3 (2.7%)	2 (2.4%)	5 (10.9%)	11 (4.0%)	
**Intraoperative tibiofibular syndesmosis repair**	**No**	25 (80.6%)	83 (76.9%)	55 (67.9%)	29 (65.9%)	192 (72.7%)	0.274
	**Yes**	6 (19.4%)	25 (23.1%)	26 (32.1%)	15 (34.1%)	72 (27.3%)	
**Intraoperative posterior malleoli management**	**No surgery**	8 (25.8%)	44 (40.7%)	38 (47.0%)	2 (4.5%)	92 (34.9%)	<0.001*
	**Lag screw**	14 (45.2%)	35 (32.4%)	26 (32.1%)	9 (20.5%)	84 (31.8%)	
	**Buttress plate**	9 (29%)	29 (26.9%)	17 (21.0%)	33 (75.0%)	88 (33.3%)	

*Statistically significant *P* < 0.05. *SA* supination-adduction, *SE* supination-external rotation, *PA* pronation-abduction, *PE* pronation-external rotation

**Fig. 3:**
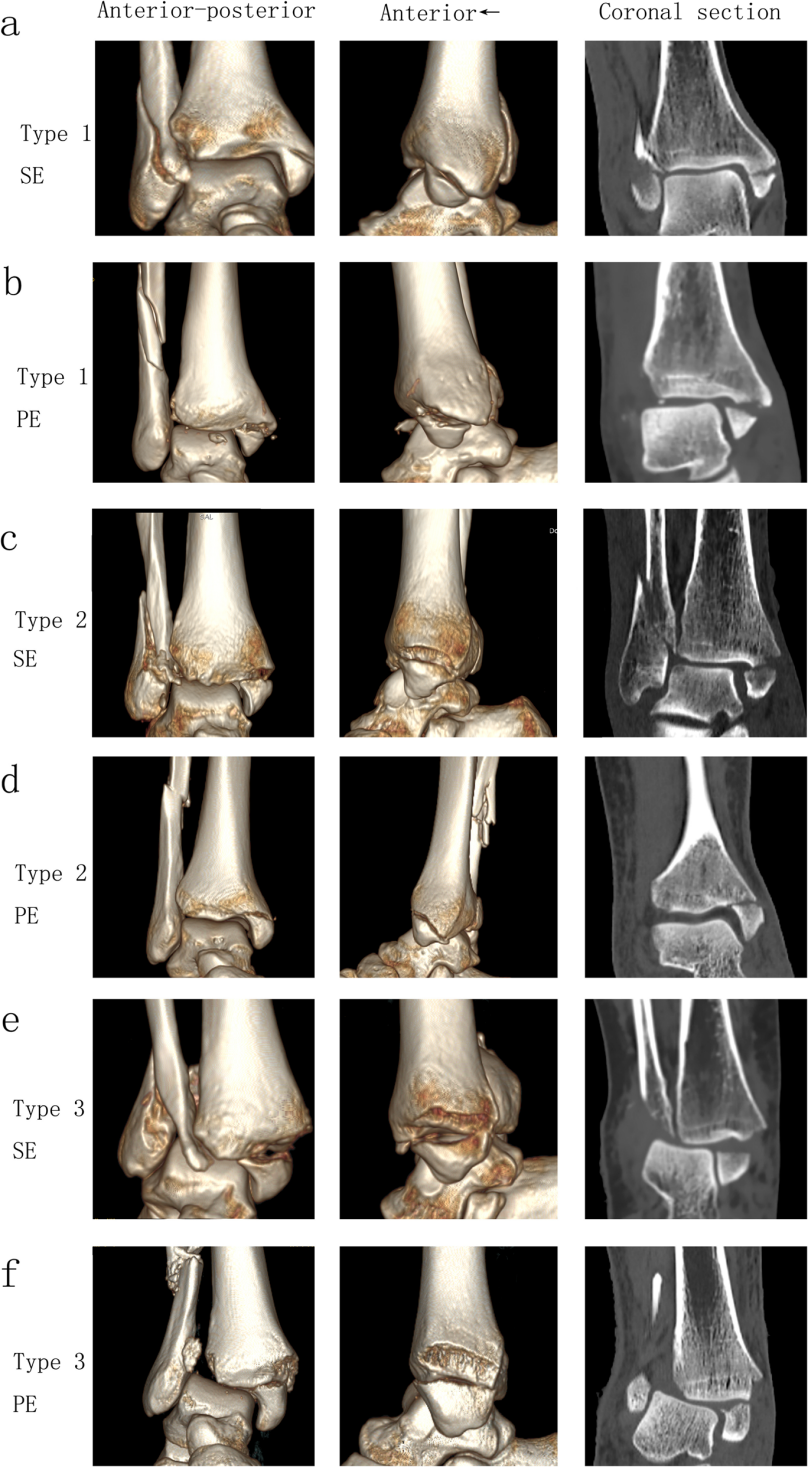
Representative 3-D reconstruction CT images of type 1/2/3 in cases of Lauge-Hansen supination-external rotation (SE) compared with pronation-external rotation (PE). **a** Type 1 of SE. **b** Type 1 of PE. **c** Type 2 of SE. **d** Type 2 of PE. **e** Type 3 of SE. **f** Type 3 of PE

Statistical analysis showed significant associations of the new classification with the Lauge-Hansen classification (*P* = 0.002) and the Danis-Weber classification (*P* = 0.012). From type 1 to type 4, the percent of Lauge-Hansen pronation-external rotation type (PE) versus supination-external rotation type (SE) was increasing and the Danis-Weber type C versus type B was increasing. Representative 3-D CT images of fracture line 1/2/3 in cases of Lauge-Hansen type SE compared with type PE are presented in Fig. 3. Although statistical analysis showed that type SE correlated with type 1 and type 2 while type PE correlated with type 3 and type 4, only 56.4% of cases conformed to the rules. Subgroup analysis by the Lauge-Hansen classification (presented in Supplement Table 1) and by the new classification (presented in Supplement Table 2) found similar results and little significance could be detected, indicating that the clinical significance of the new classification was not totally dependent on the Lauge-Hansen classification.

### The new classification showed implications in the severity of ankle fractures

From type 1 to type 4, statistical analysis showed that more and more cases could be found in the following aspects of Lauge-Hansen type PE (*P* = 0.002), the Danis-Weber type C (*P* = 0.012), comminuted medial malleolar fracture (*P* < 0.001), joint surface involvement of the distal tibial plafond (*P* < 0.001), the intraoperative posterior malleolus management (*P* < 0.001), the comminuted rates of medial malleolus fracture (*P* < 0.001), the lateral displacement of the talus (*P* < 0.001), the joint surface involvements of the distal tibial plafond (*P* < 0.001), and the angles between the major fracture line and distal tibial plafond (*P* < 0.001).

From type 1 to type 4, more and more cases could be found in the distal tibiofibular syndesmotic injury (*P* = 0.041). Although the repair rates of intraoperative tibiofibular syndesmosis were increasing, no difference could be found by statistic analysis considering the small sample size (*P* = 0.274).

### Comminuted medial malleolar fractures could be subdivided by the combination of two fracture lines

**Fig. 4:**
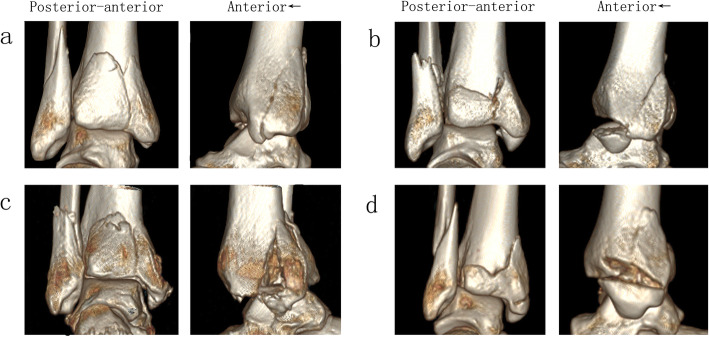
The comminuted fracture pattern of type 4. **a** Isolated fracture line 4. **b** Fracture line 4 together with fracture line 1. **c** Fracture line 4 together with fracture line 2. **d** Fracture line 4 together with fracture line 3

**Fig. 5:**
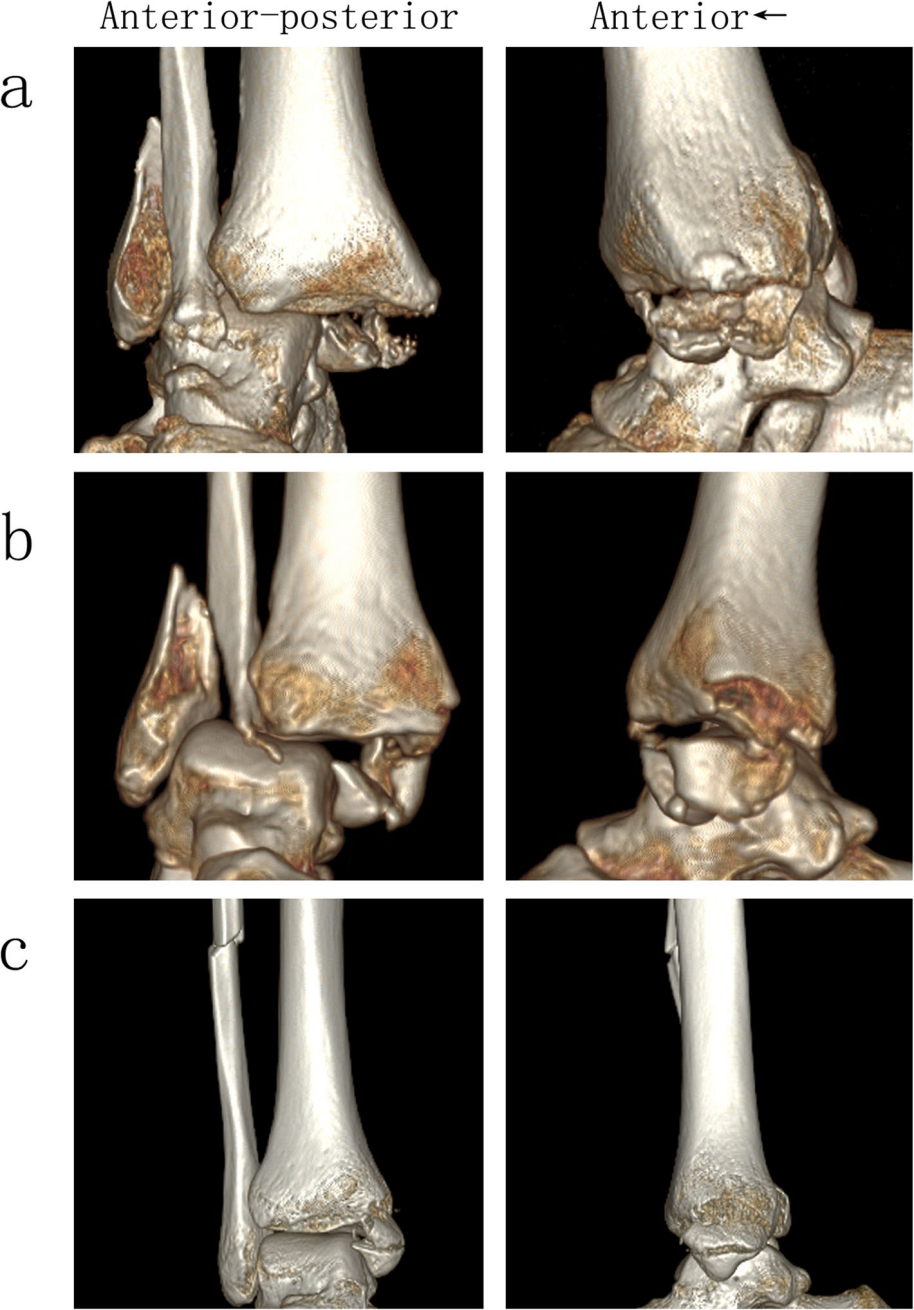
The comminuted fracture pattern of fracture line 1/2/3. **a** Fracture line 1 together with fracture line 2. **b** Fracture line 1 together with fracture line 3. **c** Fracture line 2 together with fracture line 3

The comminuted fracture patterns of medial malleolus are shown in Table 2, and all of the comminuted fractures (44/273, 16.1%) could be specified by the combination of two fracture lines (local tiny fracture fragments were excluded). The comminuted fracture pattern of fracture line 4 and its co-existence is presented in Fig. 4. The comminuted fracture pattern of fracture line 1/2/3 is presented in Fig. 5.

**Table 2:** The distribution of comminuted medial malleolus fracture by fracture lines

Fracture lines	Fracture line 1	Fracture line 2	Fracture line 3	Fracture line 4	Total
**No other fracture line**	33 (12.1%)	105 (38.5%)	73 (26.7%)	18 (6.6%)	229 (83.9%)
**Fracture line 1 co-existence**	–	5 (1.8%)	4 (1.5%)	10 (3.7%)	19 (7.0%)
**Fracture line 2 co-existence**	5 (1.8%)	–	7 (2.6%)	15 (5.5%)	27 (9.9%)
**Fracture line 3 co-existence**	4 (1.5%)	7 (2.6%)	–	3 (1.1%)	14 (5.1%)
**Fracture line 4 co-existence**	10 (3.7%)	15 (5.5%)	3 (1.1%)	–	28 (10.3%)

### Different intraoperative managements adopted according to the new classification

According to the 3-D morphology presented in Fig. 2, different surgical approaches and fixation methods were adopted in our surgeries. As indicated in Table 1, the anterior-inferior surgical approach was adopted for all the type 1/2/3 cases while the posterior-medial surgical approach was adopted for 11/46 (23.9%) cases of type 4 (*P* < 0.001). For intraoperative medial malleolar fixation methods, a significant difference was found between the four groups. Lag screws were adopted for most of the type 1/2 cases while buttress plates were adopted for some of the type 3/4 cases (*P* < 0.001). Because the 3-D reconstruction CT was routinely performed on all of the ankle fractures at our department, we had not compared the surgical decision-making based on the CT classification versus only X-ray films.

## Discussion

The study was conducted to describe a novel classification system for medial malleolar fracture based on the 3-D direction of fracture lines and centered on the posterior colliculus. We prospectively enrolled 273 cases and statistical analysis showed that the new classification had implications in the severity of ankle fractures. Furthermore, comminuted medial malleolar fractures could be subdivided, and the new classification could provide useful information for decision-making.

Several fracture classifications based on X-ray films were raised for the medial malleolar fractures in the last few years. The Pankovich system [9, 10] recognizes the location of the medial malleolar fracture and competency of the deltoid ligament and divides the fractures into 6 groups. Without the definite determination of the deltoid ligament available, the modified Pankovich classification [11] simplifies the classification system into 4 categories. The author found that the modified Pankovich type A fracture predicted supination with a 66.7% specificity and hyperplantarflexion with a 61.9% specificity, while a modified Pankovich type B fracture was 100% specific to detect pronation [11]. The Herscovici system [14] was raised to compare the functional outcomes of conservative treatment compared with surgical treatment in 57 isolated medial malleolar fractures. Little clinical significance of the classification system was mentioned [14]. Ebraheim system [13] divided 112 medial malleolar fractures into transverse, oblique, vertical, and comminuted types based on the level and location of the medial malleolar fracture. The author found that transverse fractures were the most common and correlated with type SE, oblique fractures with type PE, and vertical fractures with supination-adduction [13]. Because clinical significance was still unclear, these classifications had not been widely used by clinical surgeons.

The stability of the ankle joint relies on the integrity of the lateral and medial complexes as well as the distal tibiofibular syndesmosis [17, 18]. Especially the posterior colliculus and deep deltoid ligament, it is now believed to be the primary restraint to talar external rotation and the primary stabilizer of the medial ankle [1, 15, 19]. Considering the importance of the posterior colliculus, we raised the new fracture line classification based on the 3-D reconstruction CT. The classification integrates the Pankovich system and the Ebraheim system, and it combined the anterior/posterior colliculus fracture with vertical fracture lines. The modified Pankovich classification B was matched up with our classification of type 1, and the modified Pankovich classification D was partly matched up with our classification of type 3. From the statistical analysis, the clinical significance of the new classification could be found in the following aspects. Firstly, the new classification had significant associations with the classical ankle fracture classifications and had implications in the severity of ankle fractures. Secondly, comminuted medial malleolar fractures could be subdivided by the combination of two fracture lines. According to the combination, the comminuted fragments could then be fixed respectively during operation. Thirdly, different surgical approaches and fixation methods could be adopted according to the new classification. The posterior-medial surgical approach was only adopted for some of the type 4 cases, and buttress plates were only adopted for some of the type 3/4 cases.

The study had several limitations. Firstly, without definite information of the deltoid ligament, the important ligament was not included in the new classification. Secondly, it was a prospective observational study designed for fracture classification with no follow-up information. Thirdly, because 3-D reconstruction CT was routinely performed on all of the ankle fractures at our department, we had not compared the surgical decision-making based on the CT classification versus only X-ray classifications.

The study was conducted to describe a novel classification system for medial malleolar fracture. Based on the 3-D direction of fracture lines and centered on the posterior colliculus, we classified the medial malleolar fracture into 4 types. We prospectively enrolled 273 cases and found that the new classification had implications in the severity of ankle fractures. From type 1 to type 4, the ankle joint was more and more unstable. Furthermore, comminuted medial malleolar fractures could be subdivided, and the new classification could provide useful information for surgical decision-making.

## Supplementary Information

**Additional file 1: Supplement Table 1.** Subgroup analysis of the Lauge Hansen classification for cases in the new fracture line types. **Supplement Table 2.** Subgroup analysis of the new fracture line classification for cases in different Lauge Hansen types.

## Data Availability

All data generated during this study are included in this published article.

## Abbreviations

SA: Supination-adduction
SE: Supination-external rotation
PA: Pronation-abduction
PE: Pronation-external rotation
