# Supplementary material for: A novel classification for medial malleolar fracture based on the 3-D reconstruction CT
Source: J Orthop Surg Res. 2021 Aug 28;16:538. doi: 10.1186/s13018-021-02688-9 (PMC8400757; doi:10.1186/s13018-021-02688-9)
Supplement: Supplementary file 1 — Additional file 1: Supplement Table 1. Subgroup analysis of the Lauge Hansen classification for cases in the new fracture line types. Supplement Table 2. Subgroup analysis of the new fracture line classification for cases in different Lauge Hansen types. [file 13018_2021_2688_MOESM1_ESM.doc]

**Supplement Table 1**: Subgroup analysis of the Lauge Hansen classification for cases in the new fracture line types

| **Characteristics** | | **SE (N=206)** | | | | | | **PE (N=59)** | | | | |
| --- | --- | --- | --- | --- | --- | --- | --- | --- | --- | --- | --- | --- |
|  |  | **Type 1**  **N=30** | **Type 2**  **N=90** | **Type 3**  **N=52** | | **Type 4**  **N=34** | **P** | **Type 1**  **N=3** | **Type 2**  **N=22** | **Type 3**  **N=22** | **Type 4**  **N=12** | **P** |
| **Gender** | **Female** | 13 | 51 | | 30 | 18 | 0.588 | 1 | 7 | 12 | 6 | 0.452 |
|  | **Male** | 17 | 39 | 22 | | 16 |  | 2 | 15 | 9 | 6 |  |
| **Age (years)** | | 46±17.9 | 47.0±16.7 | | 55.0±12.2 | 48.0±15.2 | **0.016*** | 28.7±8.7 | 37.6±13.0 | 35.6±10.4 | 40.7±12.9 | 0.406 |
| **Mechanism of injury** | **Low energy** | 26 | 78 | | 39 | 27 | 0.296 | 2 | 14 | 16 | 10 | 0.675 |
|  | **High energy** | 4 | 12 | 13 | | 7 |  | 1 | 8 | 6 | 2 |  |
| **Danis-Weber classification** | **A** | 0 | 2 | | 2 | 1 | 0.433 | 0 | 0 | 0 | 0 | **0.635** |
|  | **B** | 29 | 87 | 47 | | 30 |  | 0 | 0 | 1 | 0 |  |
|  | **C** | 1 | 1 | 3 | | 3 |  | 3 | 22 | 21 | 12 |  |
| **Modified Pankovich classification** | **B** | 29 | 1 | | 0 | 0 | **<0.001*** | 3 | 0 | 0 | 0 | **<0.001*** |
|  | **C** | 1 | 1 | 0 | | 10 |  | 0 | 0 | 0 | 6 |  |
|  | **D** | 0 | 88 | 52 | | 24 |  | 0 | 22 | 22 | 6 |  |
| **Herscovici classification** | **B** | 25 | 33 | | 1 | 3 | **<0.001*** | 3 | 6 | 1 | 0 | **<0.001*** |
|  | **C** | 5 | 57 | 39 | | 9 |  | 0 | 16 | 19 | 5 |  |
|  | **D** | 0 | 0 | 12 | | 22 |  | 0 | 0 | 2 | 7 |  |
| **Anterior and posterior colliculus separation** | **No** | 3 | 88 | | 48 | 12 | **<0.001*** | 0 | 20 | 21 | 4 | **<0.001*** |
|  | **Yes** | 27 | 2 | 4 | | 22 |  | 3 | 2 | 1 | 8 |  |
| **Comminuted medial malleolus fracture** | **No** | 25 | 78 | | 35 | 9 | **<0.001*** | 3 | 17 | 16 | 2 | **0.001*** |
|  | **Yes** | 5 | 12 | 17 | | 25 |  | 0 | 5 | 6 | 10 |  |
| **Distal tibiofibular syndesmosis injury** | **No** | 26 | 67 | | 38 | 24 | 0.476 | 0 | 1 | 0 | 1 | 0.051 |
|  | **Injury** | 4 | 23 | 13 | | 10 |  | 2 | 3 | 5 | 7 |  |
|  | **Separation** | 0 | 0 | 1 | | 0 |  | 1 | 18 | 17 | 4 |  |
| **Maisonneuve fracture** | **No** | 30 | 90 | | 52 | 34 | NA | 2 | 17 | 20 | 11 | 0.423 |
|  | **Yes** | 0 | 0 | 0 | | 0 |  | 1 | 5 | 2 | 1 |  |
| **Lateral displacement of talus (mm)** | | 0.71±0.42 | 0.79±0.40 | | 0.86±0.49 | 0.54±0.36 | **0.007*** | 0.50±0.26 | 1.16±0.97 | 1.17±0.65 | 0.43±0.27 | **0.018*** |
| **Joint surface involvement of distal tibial plafond** | **No** | 30 | 87 | | 42 | 13 | **<0.001*** | 3 | 19 | 13 | 4 | **0.008*** |
|  | **Yes** | 0 | 3 | 10 | | 21 |  | 0 | 3 | 9 | 8 |  |
| **Angle between the major fracture line and distal tibial plafond (degree)** | | 7.4±12.3 | 16.4±14.0 | | 35.9±15.8 | 52.5±13.4 | **<0.001*** | -1.0±6.6 | 16.2±10.0 | 33.2±15.6 | 61.9±17.3 | **<0.001*** |
| **Intraoperative surgical approach** | **Anterior- inferior** | 30 | 60 | | 52 | 27 | **<0.001*** | 3 | 22 | 22 | 8 | **<0.001*** |
|  | **Posterior- medial** | 0 | 0 | 0 | | 7 |  | 0 | 0 | 0 | 4 |  |
| **Intraoperative medial malleolus fixation method** | **Lag screw** | 29 | 88 | | 50 | 20 | **<0.001*** | 3 | 21 | 22 | 5 | **<0.001*** |
|  | **Buttress plate** | 0 | 0 | 0 | | 11 |  | 0 | 0 | 0 | 5 |  |
|  | **K-wire** | 1 | 2 | 2 | | 3 |  | 0 | 1 | 0 | 2 |  |
| **Intraoperative tibiofibular syndesmosis repair** | **No** | 24 | 77 | | 42 | 23 | 0.373 | 1 | 6 | 7 | 6 | 0.757 |
|  | **Yes** | 4 | 10 | 9 | | 9 |  | 2 | 15 | 15 | 6 |  |
| **Intraoperative posterior malleoli management** | **No surgery** | 7 | 32 | | 21 | 1 | **<0.001*** | 1 | 12 | 10 | 1 | **0.011*** |
|  | **Lag screw** | 14 | 33 | 17 | | 8 |  | 0 | 2 | 8 | 1 |  |
|  | **Buttress plate** | 7 | 22 | 13 | | 23 |  | 2 | 7 | 4 | 10 |  |

*Statistically significant P<0.05; SE: supination-external rotation; PE: pronation-external rotation; NA: not available.

**Supplement Table 2**: Subgroup analysis of the new fracture line classification for cases in different Lauge Hansen types

| **Characteristics** | | **Type 1 (N=33)** | | | **Type 2 (N=112)** | | | **Type 3 (N=74)** | | | **Type 4 (N=46)** | | | **Total** | | |
| --- | --- | --- | --- | --- | --- | --- | --- | --- | --- | --- | --- | --- | --- | --- | --- | --- |
|  |  | **SE**  **N=30** | **PE**  **N=3** | **P** | **SE**  **N=90** | **PE**  **N=22** | **P** | **SE**  **N=52** | **PE**  **N=21** | **P** | **SE**  **N=34** | **PE**  **N=12** | **P** | **SE**  **N=206** | **PE**  **N=59** | **P** |
| **Gender** | **Female** | 13 | 1 | 0.738 | 51 | 7 | **0.037*** | 30 | 12 | 0.803 | 18 | 6 | 0.861 | 112 | 26 | 0.163 |
|  | **Male** | 17 | 2 |  | 39 | 15 |  | 22 | 10 |  | 16 | 6 |  | 94 | 33 |  |
| **Age (years)** | | 46.0± 17.9 | 28.7±8.7 | 0.111 | 47.0±16.7 | 37.6±13.0 | **0.007*** | 55.0±12.2 | 36.0±10.5 | **<0.001*** | 48.0±15.2 | 40.7±12.9 | 0.141 | 49.1±15.9 | 37.1±11.9 | **<0.001*** |
| **Mechanism of injury** | **Low energy** | 26 | 2 | 0.357 | 78 | 14 | **0.011*** | 39 | 16 | 0.838 | 27 | 10 | 0.768 | 170 | 42 | 0.055 |
|  | **High energy** | 4 | 1 |  | 12 | 8 |  | 13 | 6 |  | 7 | 2 |  | 36 | 17 |  |
| **Danis-Weber classification** | **A** | 0 | 0 | **<0.001*** | 2 | 0 | **<0.001*** | 2 | 0 | <**0.001*** | 1 | 0 | **<0.001*** | 5 | 0 | **<0.001*** |
|  | **B** | 29 | 0 |  | 87 | 0 |  | 47 | 1 |  | 30 | 0 |  | 193 | 1 |  |
|  | **C** | 1 | 3 |  | 1 | 22 |  | 3 | 21 |  | 3 | 12 |  | 8 | 58 |  |
| **Modified Pankovich classification** | **B** | 29 | 3 | 0.748 | 1 | 0 | 0.780 | 0 | 0 | NA | 0 | 0 | 0.198 | 30 | 3 | 0.094 |
|  | **C** | 1 | 0 |  | 1 | 0 |  | 0 | 0 |  | 10 | 6 |  | 12 | 6 |  |
|  | **D** | 0 | 0 |  | 88 | 22 |  | 52 | 22 |  | 24 | 6 |  | 164 | 50 |  |
| **Herscovici classification** | **B** | 25 | 3 | 0.443 | 33 | 6 | 0.407 | 1 | 1 | 0.325 | 3 | 0 | 0.409 | 62 | 10 | 0.098 |
|  | **C** | 5 | 0 |  | 57 | 16 |  | 39 | 19 |  | 9 | 5 |  | 110 | 40 |  |
|  | **D** | 0 | 0 |  | 0 | 0 |  | 12 | 2 |  | 22 | 7 |  | 34 | 9 |  |
| **Anterior and posterior colliculus separation** | **No** | 3 | 0 | 0.566 | 88 | 20 | 0.120 | 48 | 21 | 0.622 | 12 | 4 | 0.902 | 151 | 45 | 0.647 |
|  | **Yes** | 27 | 3 |  | 2 | 2 |  | 4 | 1 |  | 22 | 8 |  | 55 | 14 |  |
| **Comminuted medial malleolus fracture** | **No** | 25 | 3 | 0.443 | 78 | 17 | 0.271 | 35 | 16 | 0.786 | 9 | 2 | 0.494 | 147 | 38 | 0.305 |
|  | **Yes** | 5 | 0 |  | 12 | 5 |  | 17 | 6 |  | 25 | 10 |  | 59 | 21 |  |
| **Distal tibiofibular syndesmosis injury** | **No** | 26 | 0 | **<0.001*** | 67 | 1 | **<0.001*** | 38 | 0 | **<0.001*** | 24 | 1 | **<0.001*** | 155 | 2 | **<0.001*** |
|  | **Injury** | 4 | 2 |  | 23 | 3 |  | 13 | 5 |  | 10 | 7 |  | 50 | 17 |  |
|  | **Separation** | 0 | 1 |  | 0 | 18 |  | 1 | 17 |  | 0 | 4 |  | 1 | 40 |  |
| **Maisonneuve fracture** | **No** | 30 | 2 | **0.001*** | 90 | 17 | **<0.001*** | 52 | 20 | 0.086 | 34 | 11 | 0.089 | 206 | 50 | **<0.001*** |
|  | **Yes** | 0 | 1 |  | 0 | 5 |  | 0 | 2 |  | 0 | 1 |  | 0 | 9 |  |
| **Lateral displacement of talus (mm)** | | 0.71± 0.42 | 0.50±0.26 | 0.410 | 0.79±0.40 | 1.16±0.97 | 0.095 | 0.86±0.49 | 1.21±0.64 | 0.029* | 0.54±0.36 | 0.43±0.27 | 0.296 | 0.75± 0.43 | 0.98±0.78 | **0.004*** |
| **Joint surface involvement of distal tibial plafond** | **No** | 30 | 3 | NA | 87 | 19 | 0.054 | 42 | 13 | 0.051 | 13 | 4 | 0.762 | 172 | 39 | **0.003*** |
|  | **Yes** | 0 | 0 |  | 3 | 3 |  | 10 | 9 |  | 21 | 8 |  | 34 | 20 |  |
| **Angle between the major fracture line and distal tibial plafond (degree)** | | 7.4± 12.3 | -1.0±6.6 | 0.259 | 16.4±14.0 | 16.2±10.0 | 0.968 | 35.9±15.8 | 33.1±15.9 | 0.512 | 52.5±13.4 | 61.9±17.3 | 0.128 | 24.3± 20.0 | 28.7±21.4 | 0.154 |
| **Intraoperative surgical approach** | **Anterior- inferior** | 30 | 3 | NA | 90 | 22 | NA | 52 | 22 | NA | 27 | 8 | 0.374 | 199 | 54 | 0.239 |
|  | **Posterior- medial** | 0 | 0 |  | 0 | 0 |  | 0 | 0 |  | 7 | 4 |  | 7 | 4 |  |
| **Intraoperative medial malleolus fixation method** | **Lag screw** | 29 | 3 | 0.748 | 88 | 21 | 0.545 | 50 | 22 | 0.351 | 20 | 5 | 0.548 | 187 | 51 | 0.606 |
|  | **Buttress plate** | 0 | 0 |  | 0 | 0 |  | 0 | 0 |  | 11 | 5 |  | 11 | 5 |  |
|  | **K-wire** | 1 | 0 |  | 2 | 1 |  | 2 | 0 |  | 3 | 2 |  | 8 | 3 |  |
| **Intraoperative tibiofibular syndesmosis repair** | **No** | 24 | 1 | 0.072 | 77 | 6 | **<0.001*** | 42 | 7 | **<0.001*** | 23 | 6 | 0.266 | 166 | 20 | **<0.001*** |
|  | **Yes** | 4 | 2 |  | 10 | 15 |  | 9 | 15 |  | 9 | 6 |  | 32 | 38 |  |
| **Intraoperative posterior malleoli management** | **No surgery** | 7 | 1 | 0.309 | 32 | 12 | 0.095 | 21 | 10 | 0.827 | 1 | 1 | 0.449 | 8 | 1 | 0.070 |
|  | **Lag screw** | 14 | 0 |  | 33 | 2 |  | 17 | 8 |  | 8 | 1 |  | 61 | 24 |  |
|  | **Buttress plate** | 7 | 2 |  | 22 | 7 |  | 13 | 4 |  | 23 | 10 |  | 65 | 23 |  |

*Statistically significant P<0.05; SE: supination-external rotation; PE: pronation-external rotation; NA: not available.
